# Poly(ADP-Ribose)Polymerase-1 in Lung Inflammatory Disorders: A Review

**DOI:** 10.3389/fimmu.2017.01172

**Published:** 2017-09-19

**Authors:** Gurupreet S. Sethi, Vivek Dharwal, Amarjit S. Naura

**Affiliations:** ^1^Department of Biochemistry, Panjab University, Chandigarh, India

**Keywords:** asthma, acute lung injury, chronic obstructive pulmonary disease, lung inflammation, poly(ADP-ribose)polymerase, NF-κB, STAT-6

## Abstract

Asthma, acute lung injury (ALI), and chronic obstructive pulmonary disease (COPD) are lung inflammatory disorders with a common outcome, that is, difficulty in breathing. Corticosteroids, a class of potent anti-inflammatory drugs, have shown less success in the treatment/management of these disorders, particularly ALI and COPD; thus, alternative therapies are needed. Poly(ADP-ribose)polymerases (PARPs) are the post-translational modifying enzymes with a primary role in DNA repair. During the last two decades, several studies have reported the critical role played by PARPs in a good of inflammatory disorders. In the current review, the studies that address the role of PARPs in asthma, ALI, and COPD have been discussed. Among the different members of the family, PARP-1 emerges as a key player in the orchestration of lung inflammation in asthma and ALI. In addition, PARP activation seems to be associated with the progression of COPD. Furthermore, PARP-14 seems to play a crucial role in asthma. STAT-6 and GATA-3 are reported to be central players in PARP-1-mediated eosinophilic inflammation in asthma. Interestingly, oxidative stress–PARP-1–NF-κB axis appears to be tightly linked with inflammatory response in all three-lung diseases despite their distinct pathophysiologies. The present review sheds light on PARP-1-regulated factors, which may be common or differential players in asthma/ALI/COPD and put forward our prospective for future studies.

## Introduction

Pulmonary inflammatory diseases, including asthma, chronic obstructive pulmonary disease (COPD), and acute lung injury (ALI) constitute a huge socio-economic burden on present society. Asthma alone affects around 300 million people globally, which include both the elderly and young individuals (1). About 14% children across the globe experience symptoms of the disease (2). Asthma is a chronic inflammatory disease and patients present episodic symptoms, such as wheezing, cough, chest tightness, and shortness of breath. Owing to the inflammation, inhaled corticosteroids and long-acting β_2_ agonists remain the major therapies available for the disease. Although the majority of asthmatics respond well to steroids, the treatment is still ineffective in 5–10% of the patients (3). Moreover, the long-term use of steroids is associated with numerous side effects (4, 5). Similarly, COPD is also a chronic disease characterized by persistent and usually progressive airflow limitation along with an enhanced inflammatory response (6). Currently, it is the fourth leading cause of worldwide deaths and is projected to become the third highest by 2020 (6, 7). However, there are only a limited number of pharmacological therapies available (8–11). Surprisingly, the steroids, which act as potent anti-inflammatory agents in asthma, have been found to be less effective in controlling COPD-associated inflammation (12–14). Till date, no drug that actually modifies the progressive nature of the disease is available. In contrast to asthma and COPD, ALI is an acute inflammatory disease of the lung. It is diagnosed based on its acute onset, pulmonary inflammation, increased vascular permeability, and hypoxemia (15). Data from the developed world show that the incidence of ALI varies from 10.1 to 86.2 cases per 100,000 person per year (16). Despite the advances in treatment modalities, the mortality rate in patients with ALI is still 36–44% (17). The currently available therapies include lung-protective ventilation, high-frequency oscillatory ventilation, conservative fluid strategies, and prone positioning that only mitigate the symptoms of the disease. So far, there is no pharmacotherapy available to cure the disease. Therefore, alternative therapeutic targets need to be identified for effective management/treatment of asthma, ALI, and COPD.

Poly(ADP-ribose)polymerases (PARPs) constitute an 18-member family of proteins that play an important role in DNA repair (18, 19). In addition, a more intricate physiological role of PARPs in fundamental cellular processes such as chromatin remodeling, transcription, and regulation of the cell cycle have been reported (20–22). Various studies have shown that excessive reactive oxygen species (ROS)/reactive nitrogen species (RNS) induced DNA damage that occurs during inflammatory conditions leads to an over activation of PARPs (23–26). Extensive activation of PARPs results in energy crisis due to depletion of their substrate, i.e., nicotinamide adenine dinucleotide (NAD^+^), thus leading to necrosis (27). In addition, PARPs interact with various cellular proteins and transcription factors that aid inflammation (28–33). In the last decade, significant research has been conducted to evaluate the role of PARP-1 in lung inflammation associated with asthma and ALI, using experimental models. Also, some reports hint at potential connection between PARP-1 and COPD. Given the fact that inflammation plays a critical role in these diseases, we have tried to underline the cellular and molecular factors that are regulated by PARP-1 in the orchestration of inflammation. We hope that positioning PARP-1 in context of lung inflammation associated with asthma, ALI, and COPD would provide better understanding and might enable us to develop new drugs in the area.

## PARP-1 and Its Pro-Inflammatory Role

Poly(ADP-ribose)polymerase-1 belongs to a family of 18 proteins exhibiting mono(ADP-ribosyl) or poly(ADP-ribosyl) transferase activities. Mammalian PARP-1 is a 116-kDa protein which comprises of an *N*-terminal DNA-binding domain, a nuclear localization sequence (NLS), a central automodification domain, and a C-terminal catalytic domain (Reviewed comprehensively) (34). The C-terminal region is the most conserved part of the PARPs family. It executes catalytic function by synthesizing poly(ADP)ribose (PAR) using NAD^+^ as a substrate (35, 36) and transferring such PAR moieties to several proteins, including histones, DNA repair proteins, and transcription factors resulting in their covalent modification (18, 37). Such post-translational modifications ultimately alter the structure and functions of the acceptor proteins. Under genotoxic stress conditions, PARP-1 binds itself to the nucleosomes containing intact (38) as well as damaged DNA structures (e.g., nicks and double-strand breaks); thus, leading to the activation of DNA repair enzymes (39). The covalently attached PAR can be hydrolyzed to free PAR or mono(ADP-ribose) by PAR glycohydrolase (PARG) (40). Synthesis and degradation of PAR chains is tightly controlled *in vivo* and PAR residues have a very short half-life in the cell (few minutes) (34). Free or protein-bound PAR polymers also work as signal transducers by binding other proteins (41, 42).

**Figure 1:**
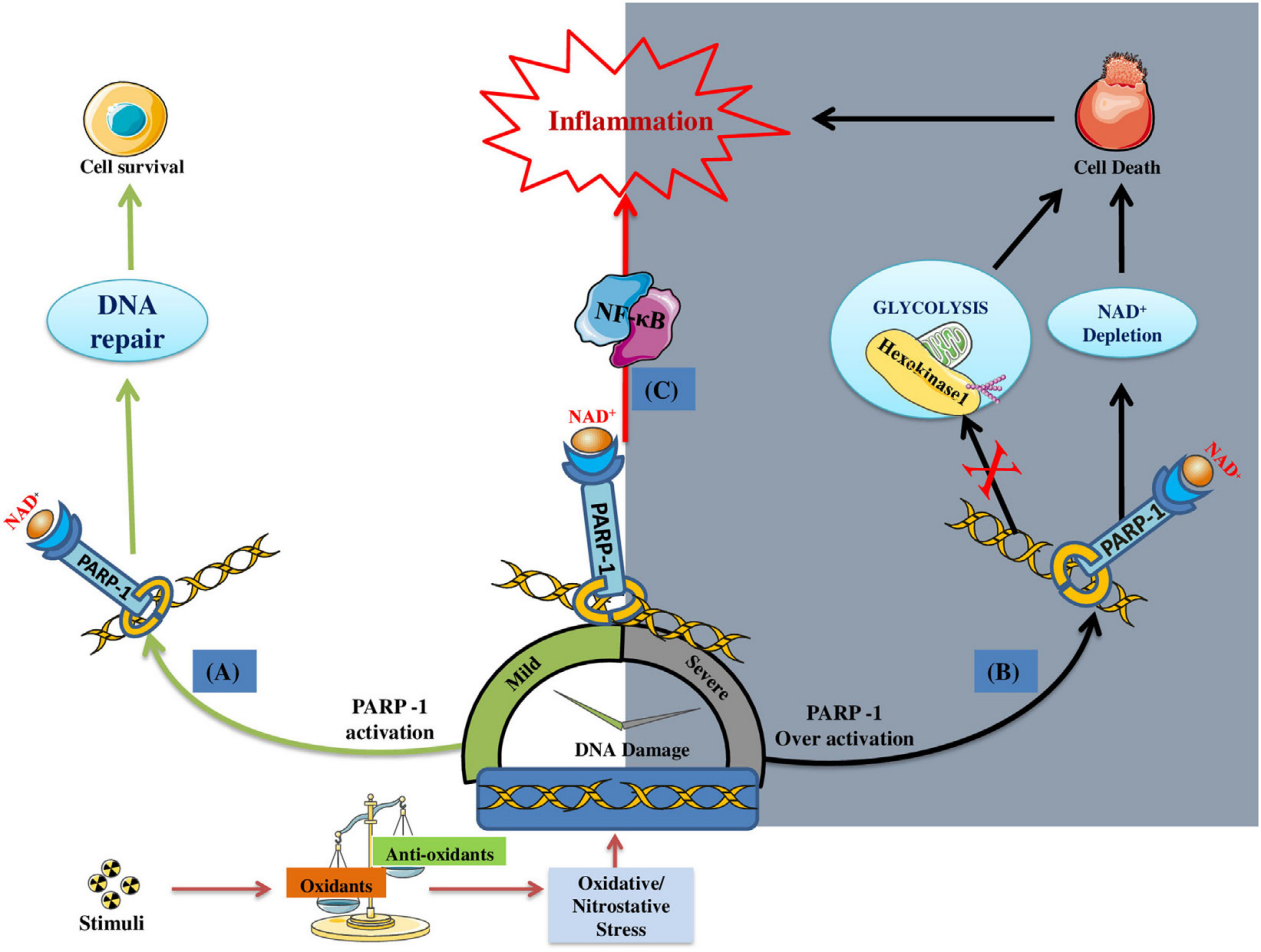
DNA damage induced poly(ADP-ribose)polymerase-1 (PARP-1) activation and its consequences on the cell fate/inflammation: **(A)** Under mild DNA damage conditions PARP-1 facilitates DNA repair; thus, promoting the survival of cell (indicated by green colored arrows). **(B)** Under severe DNA damage conditions, PARP-1 become over-active, thus causing cell death by either NAD^+^ dependent (i.e., ATP depletion/necrosis) or independent (i.e., glycolysis inhibition) manner (indicated by black colored arrows). **(C)** In addition, PARP-1 plays a pro-inflammatory role by regulating the expression of inflammatory genes through NF-κB activation (indicated by red arrow).

It is quite clear that PARP-1 gets activated in response to DNA damage induced by ROS/RNS under inflammatory conditions (43, 44) Although, the primary aim of PARP-1 is to maintain the genome integrity but its over activation under extensive and persistent DNA damaging environment promote inflammatory conditions. As stated earlier, over activation of PARP-1 depletes its substrate, i.e., NAD^+^, bringing the cell to an energy deficient state, thus leading to necrosis (45). Recently, PARP-1 has been reported to cause cell death by suppressing the activity of hexokinase-1 (an essential enzyme of glycolysis), through its posttranscriptional modification (by adding PAR chains) (46). Apart from inducing cellular death, PARP-1 has been reported to promote inflammation by influencing chromatin remodeling and expression of several pro-inflammatory factors. Since the DNA is negatively charged, poly(ADP)ribosylation (also negatively charged) of histones results in relaxing of nucleosomal structures and, hence, aids the transcription of pro-inflammatory genes (47, 48). PARP-1 regulates the expression of several NF-κB-dependent cytokines, chemokines, adhesion molecules, inducible nitric-oxide synthase (*iNOS*), required for the manifestation of inflammatory cycle (49–55). It is quite evident that PARP-1 gene deletion or its pharmacological inhibition results in suppressed migration of leukocytes to the inflammatory sites (56). Overall, PARP-1 plays a pro-inflammatory role by inducing cellular death and upregulating the expression of various inflammatory genes, *via* interaction with NF-κB (28, 29). Figure 1 gives an overview of the role of PARP-1 in inducing inflammatory conditions under stress environment.

Numerous review articles have highlighted the pro-inflammatory role of PARP-1 in various extra-pulmonary inflammatory diseases, including sepsis, arthritis, atherosclerosis, diabetic nephropathy, allergic encephalomyelitis (EAE), and contact hypersensitivity (43, 56–61). Importantly, PARP-1 inhibitors have already entered the clinical phase for testing their therapeutic potential in different types of cancers (62–64). In addition, encouraging results have been reported in a clinical trial involving patients with ST elevation myocardial infarction which is the most severe form of heart attack. Results show that pharmacological inhibition of PARP-1 with INO-1001 reduced plasma levels of C-reactive proteins (CRP) and IL-6, which are well-known inflammatory markers. Furthermore, no serious adverse effect associated with the drug was noticed (65). Based on these, it would be logical to test such FDA-approved PARP-1 inhibitors in other human inflammatory diseases in order to shorten our journey from bench to bedside in search of new therapeutic agents. Therefore, we reviewed the role PARP-1 in the context of asthma, ALI, and COPD with a special focus on inflammation.

## PARP-1 in Asthma

Asthma is a chronic inflammatory lung disorder characterized by airway inflammation, hyper-reactivity, and remodeling (66). The disease is recognized as Type-I hypersensitivity disorder (allergic disease), and various mediators, such as immune cells (T lymphocytes, eosinophils, macrophages, and monocytes), structural cells (epithelial cells, endothelial cells, and smooth muscle cells), cytokines, and transcription factors play an important role in its establishment (66, 67). Since the susceptible individuals are repeatedly exposed to allergens, recurring allergic attacks lead to the development of persistent inflammatory conditions in the lungs. Excessive production of ROS/RNS by inflammatory cells induces DNA damage and, consequently, results in increased activity of PARP-1 (32, 68, 69).

In 2003, Boulares et al. first reported that ROS-mediated DNA damage results in PARP-1 activation in the lungs of ovalbumin (OVA) exposed mice. Furthermore, it was discovered that PARP-1 inhibition (pharmacological or genetic) prevented OVA-induced lung inflammation (32). Similar results were reported by Suzuki et al. in a study conducted using guinea pig model of asthma (70). Oumouna et al. later identified that PARP-1 promotes asthma-associated inflammation by influencing the expression of Th2 cytokines, primarily IL-5 (71). In a follow-up study, we identified that PARP-1 activity was a requisite for maintaining STAT-6 integrity, which subsequently controls expression of GATA3 and IL-5 (72). Also, we observed that PARP-1 contributes toward both airway inflammation and airway hyper-reactivity (AHR) (33, 73). In addition, PARP-1 activity has been reported to be required for splenic CD4^+^ T cells maturation and the inhibition of PARP-1 results in marked increase in T regulatory (Treg) cells (73). Recently, we have reported that PARP-1 inhibition blocks asthma-like traits in mice when exposed to house dust mite (HDM) which is an important human allergen (74). Apart from the aforementioned preclinical evidences, we have demonstrated that enhanced PARP-1 activity correlates with asthma phenotype in humans. Analysis of the lung specimens and peripheral blood mononuclear cells (PBMCs) derived from healthy and asthmatic patients showed that PARP-1 indeed is activated substantially in asthmatics (74). Besides, other recent studies have indicated a role of PARP-1 in airway remodeling traits possibly by modulating the function of structural cells that line the airways (33, 73). Given the multifaceted role of PARP-1 in asthma, we have discussed its role in immune cells and structural cells along with the signaling mechanism influenced by the protein in the following section.

### Role of PARP-1 in Asthma-Associated Immune Cell Recruitment and Functioning

Different immune cells, such as CD4^+^ T cells, eosinophils, and dendritic cells (DCs), play a critical role in orchestrating asthma-associated inflammation (75, 76). Large numbers of reports confirm the active involvement of PARP-1 in modulating recruitment and functioning of these cells as discussed below.

#### PARP-1 and Lymphocytes

The CD4^+^ T cells [also referred to as T helper (Th) cells] play an important role in asthma pathogenesis (76, 77). In fact, the disease is classically known as Th2 cell*-*associated inflammatory disorder as it is marked with the increased presence of these cells in the airways of patients. Th2 cells are the primary source for cytokines, such as IL-4, IL-5, and IL-13, and are responsible for antigen-induced AHR and pulmonary eosinophilia (78, 79). Interestingly, we have recently observed that PARP-1 activity modulates splenic CD4^+^ T cell subpopulation. It was noticed that PARP-1 inhibition (genetically or pharmacologically) prevents the increase in the splenic CD4^+^ T cells in OVA-challenged mice. Such reduction in CD4^+^ T cells was associated with declined production of Th2 cytokines; eotaxin, IL-4, IL-5, and IL-13 as expected. In addition, (pharmacological inhibitor of PARP) downregulates the CD3/CD28-induced expression of GATA-3 and IL-4 mRNA in CD4^+^ T cells skewed toward Th2 phenotype. Furthermore, adoptive transfer of Th2-skewed OT-II CD4^+^T cells was found to be sufficient to reverse Th2 cytokines production in OVA exposed PARP-1^−/−^mice (73). We extended our findings by stimulating the T-cell receptor (TCR) of CD4^+^ T cells derived from humans (isolated from PBMCs of healthy subjects) and our data indeed confirm that PARP-1 inhibition suppresses the production of Th2 cytokines and expression of GATA-3 in cells of human origin (74).

Interestingly, the percentage of Treg cells was found to be increased upon PARP-1 inhibition. Such increase in splenic Treg cell population might be an added protective trait in controlling asthma-associated inflammation by PARP-1 inhibition (73). Zhang et al. studied the interaction of PARP-1 with TGF-β signaling in CD4^+^ T cells and observed that it regulates TGF-β signaling by altering the expression of its receptors, i.e., TβRs type II and I. It was reported that PARP-1 inhibits the expression of TβRII by binding directly to its promoter region and that of TβRI through its enzymatic activity. Indeed, PARP-1 inhibition enhances the expression of TβRs and activation of TGF-β signaling, which directs the binding of Smad3 at enhancer of FOXP3 gene and, consequently, increases its expression (80). The FOXP3 gene is essential for the differentiation and functioning of Treg cells (81). In addition, a direct interaction between PARP-1 and FOXP3 has been reported in another study. It was found that PARP-1 activity results in poly(ADP-ribosyl)ation of FOXP3, which reduces its stability and regulatory function. Certainly, the inhibition of PARP-1 enhances the stabilization of FOXP3 and consequent expression of its dependent genes required for immune-suppressive function of Treg cells (82).

CD4^+^ cells can also differentiate into Th17 cells through the expression of RAR-related orphan receptor γ (ROR-γt) and ensuing production of IL-17 (83). It is important to mention that IL-17 has been linked with severe asthma given its role in the recruitment of neutrophils (84, 85). Several recent studies using animal models of inflammatory diseases and in *in vitro* systems have shown conflicting findings on the relationship between PARP-1 and IL-17. Whereas some reports show an increase in IL-17 production (80), others showed no change (86) or a decrease in the cytokine upon PARP-1 inhibition (87). Therefore, we examined the effect of PARP-1 on IL-17 expression using experimental model(s) relevant to asthma. We observed that PARP-1 gene deletion results in an increased production of IL-17 upon TCR stimulation of CD4^+^ cells. Repeated administration of PARP inhibitor Olaparib in chronic HDM-exposed mice resulted in modest increase in BALF IL-17 levels but, no such increase was observed in HDM-exposed PARP-1^−/−^ mice. Overall, our data did not lead us to any concrete conclusion, as IL-17 increase observed *in vitro* system was not consistent with our results obtained using animal model. Nevertheless, we noticed that the increase in levels of IL-17 was accompanied by a complete abrogation of BALF neutrophilia upon PARP-1 inhibition either by Olaparib or gene deletion in HDM-treated mice. Furthermore, such increase in IL-17 levels coincided with a significant increase in the percentage of Treg cell population in spleens of HDM-exposed animals; thus, underlines the dual role of PARP-1 in differentiation of Treg and Th17 cells (74). It is possible that differentiation of CD4^+^ T cells toward Th17 phenotype may be a counter-regulatory action to control immune-suppressive function of Treg cells upon PARP-1 inhibition. These results suggest that the relationship between IL-17, its effects, and PARP-1 is complex and requires further investigations.

#### PARP-1 and Eosinophils

Eosinophils are granulocytes and their number is increased in air spaces and sputum of asthmatics. Thus, they are used as an important marker of the disease (88, 89). The clonal expansion of Th2 cells, during the disease development is responsible for eosinophilia. Th2 cells are source for cytokines, such as IL-4, IL-5, and GMCSF, which facilitate eosinophil recruitment and survival (90). A number of studies have shown that PARP-1 inhibition reduces the asthma-associated eosinophilia (71, 91, 92). We also found that PARP-1 inhibition (genetic or pharmacological) prevents the infiltration of eosinophils into the airways of OVA-challenged mice specifically by the suppression of IL-5 (71). In addition, we have reported that PARP-1 modulates the expression of ICAM-1, a key factor known to facilitate trans-diapedesis of leukocytes into the airways (93). There is a strong possibility that PARP-1 inhibition modulates the recruitment of eosinophils in the lung by altering expression of the Th2 cytokines as well as adhesion molecules.

#### PARP-1 and DCs

Dendritic cells are primary antigen-presenting cells (APCs) that are present throughout the pulmonary tract. They process and present foreign antigens to CD4^+^ T cells, and polarize them toward Th1, Th2, or T regulatory cell type (75, 94) and, thus, decide the nature of immune response. Interestingly, an *in vitro* study conducted by Aldinucci et al. showed that the increased PARP-1 activity is a requisite for the differentiation of human monocytes to DCs. They observed that PARP-1 inhibition [by Penanthridinone/thieno[2, 3-c]isoquinolin-5-one (TIQ-A)] reduces the expression of DC specific markers CD86 and CD83 in monocytes isolated from PBMCs when exposed to lipopolysaccharide (LPS). In addition, PARP-1 modulates the function of DCs, as inhibition of its enzymatic activity reduces the T cell allostimulatory activity of matured DCs (95). In a separate study, it was discovered that PARP-1 inhibition adversely regulates the maturation and functioning of DCs derived from mouse bone marrow. Furthermore, the effects of PARP-1 on the functioning of DCs were analyzed in mouse model of encephalomyelitis and data confirmed that PARP-1 inhibition reduces the DC number as well as their APC function (96). It is noteworthy that both the aforementioned studies were not conducted in the context of asthma. Considering the role of DCs in asthma-associated antigen presentation, more studies using classical models of experimental asthma may be required for a better conclusion.

### PARP-1 and Structural Cells

Airway remodeling is a key pathogenic feature of asthma that results in irreversible airway obstruction (97). It occurs because of persistent inflammatory conditions, recurring bronchial epithelium damage, and deregulated repair mechanism (98). Airway remodeling is marked with changes, such as, detachment of epithelial cells, sub-epithelial fibrosis, goblet-cell hyperplasia and metaplasia, airway smooth muscle thickening, and angiogenesis (99–104). Contradictory reports are available in literature regarding the role of PARP-1 in asthma-associated airway remodeling. Havranek et al. reported that PARP-1 activity is associated only with OVA-induced airway inflammation in mice but not with goblet-cell metaplasia (GCM), a characteristic feature of airway remodeling (105). It was observed that a single intratracheal instillation of IL-13 results in manifestation of GCM in mice lungs without altering the PARP-1 activity, thus suggesting PARP-1 activity is not necessary for IL-13-mediated GCM. In contrast to the aforementioned study, there are reports suggesting the protective effects of PARP-1 inhibition against allergen-induced mucus production and AHR in animal models of asthma (33, 73). Recently, it was reported that HYDAMTIQ, a selective PARP inhibitor, significantly attenuated lung inflammation as well as features of airway remodeling in OVA-challenged guinea pigs (92). However, this study highlights the initial phase of airway remodeling, as the guinea pigs underwent only two OVA challenges (once a week for two consecutive weeks), which is not sufficient to develop all the aspects of airway remodeling. In another study, Zaffini et al. evaluated the therapeutic effects of PARP-1 inhibition on airway inflammation and remodeling using HDM-induced mouse model of chronic asthma (established by challenging the mice for 5 days/week for five consecutive weeks). They observed that PARP-1 inhibition affects airway remodeling partly as sub-epithelial collagen deposition was reduced; however, α-smooth muscle actin (α-SMA) thickening remained unaffected (106). It is important to mention that IL-5 mediated eosinophil production can lead to airway remodeling through the release of TGF-β, a pro-fibrotic factor (88, 107). However, it is not clear whether PARP-1 inhibition modulates the TGF-β/IL-13 linked pro-fibrotic signaling mechanism(s) in structural cells (such as epithelial, fibroblasts, or smooth muscle cells) or suppression in airway remodeling features is simply an outcome of lesser TGF-β release due to reduced eosinophil number.

Epithelial–mesenchymal transition (EMT) is an important phenomenon that is associated with airway remodeling in chronic asthma. During EMT, epithelial cells lose their characteristics (such as polarity as well as cell–cell adhesion) and acquire features of mesenchymal cells (like becoming migratory and secretory) (108). Different studies have shown that TGF-β plays a crucial role in EMT. TGF-β is known to downregulate the expression of E-cadherin (epithelial cell marker) and upregulate the expression of N-cadherin, fibronectin (FN-1), vitronectine, and MMPs (mesenchymal cell marker) *via* activation of snail (a transcriptional repressor of epithelial genes) (108, 109). In addition, participation of NF-κB in EMT is reported (110). In epithelial cells, p65 subunit of NF-κB is associated with E-cadherin. During EMT, levels of E-cadherin are reduced, which freed the p65 to form dimer complex(s) with other NF-κB subunits. NF-κB then occupies the promoters of various mesenchymal genes for their enhanced expression (111). Stanisavljevic et al. have reported that PARP-1 plays an important role in the formation and binding of ternary complex (along with Snail1 and p65 NF-κB) to the FN-1 promoter region for its efficient expression (112). It is possible that PARP-1 regulates airway remodeling in chronic asthma partly by modulating EMT process.

### PARP-1-Associated Signaling Pathways in Modulating Asthma-Associated Cytokines Production

A vast network of cytokines, primarily Th2 cytokines, is known to regulate asthma-associated inflammation. Among the different members of Th2 cytokines family, IL-4, IL-5, and IL-13 contribute primarily in asthma (113). IL-4 is responsible for expansion and activation of Th2 lymphocytes, activation of B cells to produce antigen specific IgE, and stimulation of IL-5 expression. IL-5 participates in the recruitment, activation, and degranulation of eosinophils in the lungs of an individual exposed to allergen (114–118). IL-13 plays an important role in airway remodeling as it contributes in IgE synthesis, mucus hyper secretion, and fibrosis (119–121).

Different studies have been carried out by our research group to evaluate the role of PARP-1 in modulating asthma-associated cytokines production. We have reported that PARP-1 inhibition (genetic or pharmacological) significantly reduces the eosinophil infiltration into the airway of OVA-challenged mice. Interestingly, eosinophilia was reversed in lungs of PARP-1^−/−^ mice upon intranasal replenishment of IL-5 but not IL-4 or IgE, which indicates a direct regulatory relationship between PARP-1 and IL-5 (71). We further elucidated the potential mechanism by which PARP-1 regulates the IL-5 expression during allergic conditions. Analysis of the various steps in IL-5 production revealed that PARP-1 modulates the expression of IL-5 at mRNA level. PARP-1 inhibition downregulates IL-5 expression by promoting STAT-6 degradation without alerting JAK1/JAK3 activation (72). In addition, we observed that PARP-1 gene deletion significantly increases the production of Th1 cytokines (IL-2 and IL-12, but not IFN-γ) in OVA-restimulated splenocytes derived from OVA-challenged PARP-1^−/−^ mice. Overall, PARP-1 gene deletion tilts the Th1/Th2 balance toward Th1 in favor of anti-allergic response (71).

Different transcription factors, such as STAT-6, GATA-3, activator protein 1 (AP-1), and NF-κB, are involved in the pathogenesis of asthma (122). Among them, STAT-6 is involved in the development of Th2 cell-mediated immune response, immunoglobulin (Ig) class switching (IgE and IgG1 in B cells), mucus production (in lung epithelial cells), cell contractility, and eotaxin production (in smooth muscle cells), as well as regulation of GATA-3 expression (a master regulator of Th2 cells differentiation) (123–128). Our data indicate that PARP-1 activity is one of the requisites for maintaining the integrity of STAT-6. It was observed that PARP-1 inhibition downregulated the expression of STAT-6 at the protein level without altering its gene expression. Lack of STAT-6 stability results in reduced expression of GATA-3 and its dependent gene, IL-5. Overall, PARP-1 seems to modulate recruitment of eosinophils in the lungs of allergen exposed animals by influencing the STAT-6–GATA-3–IL5 axis (72).

Poly(ADP-ribose)polymerase-14, another member of the PARP family with intracellular mono-ADP-ribosyltransferase activity, has been shown to be associated with asthma pathogenesis. PARP-14 is expressed in lymphoid organs and lymphocyte cell lines and plays an important role in the signal transduction (129, 130). Mehrotra et al. have shown that PARP-14 acts as a transcriptional switch for STAT-6-dependent gene induction. In the absence of IL-4, PARP-14 acts as a transcriptional repressor by recruiting histone deacetylases (HDACs) (HDACs 2 and 3) to IL-4 responsive promoters. By contrast, in the presence of IL-4, the catalytic activity of PARP-14 facilitates the release of HDACs, which allows the binding of STAT-6 to the promoter region of its target genes, thereby activating STAT-6-dependent transcription (131). In a subsequent study by the same group, the role of PARP-14 in OVA-induced lung inflammation and reactivity was analyzed in mice. It was reported that PARP-14 participates in differentiation of naive T cells toward Th2 phenotype without affecting Th1 cytokine INF-γ. PARP-14 gene deletion leads to a substantial decrease in IgE and Th2 cytokines levels. In addition, it was found that pharmacological inhibition of PARP-14 during sensitization or challenge stage ameliorated the asthmatic inflammation effectively (132). These aforementioned studies indicate that other members of the PARP family (besides PARP-1) might play a role in asthma pathogenesis. Considering the structural and functional homology among the different members of PARP family, more studies are required for better understanding.

In addition to the involvement of PARP-1 in modulating the STAT-6/GATA-3 mediated trans-activation, several studies suggest the critical role of PARP-1 in the activation of NF-κB, a redox-sensitive transcription factor. It is well established that NF-κB regulates the transcription of a wide array of gene products (cytokines, chemokines, adhesion molecules, growth factors, etc.) that are involved in the molecular pathobiology of the lung (133, 134). It has been reported that PARP-1 secures its position upstream of NF-κB signaling pathway as it is required for the activation of IKK and NF-κB (135, 136). Hassa et al. have reported that neither the DNA binding nor the enzymatic activity of PARP-1 is required for the activation of NF-κB. However, they found that direct protein–protein interaction of PARP-1 with both subunits of NF-κB is required for activation of NF-κB (137). In contrast to this, Liu et al. have found that enzymatic activity of PARP-1 is required for activation of NF-κB in LPS-stimulated murine macrophage (138). We also have observed that PARP-1 activity might be responsible for the retention of NF-κB in nucleus as PARylation of p65 NF-κB subunit decreases its interaction with Crm1, a nuclear exporting protein (28). The dependency of NF-κB on PARP-1 for its efficient activation correlated well under asthmatic conditions, as nuclear localization of p65NF-κB was also found to be defective in lung tissue sections derived from PARP-1^−/−^ mice given allergen exposure (49). Overall, there is strong evidence that PARP-1 modulates NF-κB activation directly or indirectly. Based on the fact that NF-κB signaling is influenced by oxidant/antioxidant imbalance in airways (139, 140) and the reported role of transcription factor on the expression of GATA-3 and subsequent Th2 differentiation in allergic airway inflammation (141), it is highly plausible that PARP-1 plays a central role in asthmatic airway inflammation potentially through activation of NF-κB. As multiple signal transduction pathways in inflammation converge on the NF-κB complex, various strategies targeting NF-κB signaling are being pursued for asthma treatment. It is important to mention that therapeutics that target NF-κB/IκBα complex dissociation or NF-κB/DNA binding has the potential to treat even steroid-insensitive airway disease.

Activator protein 1 is another transcriptional factor that plays an important role in the pathogenesis of asthma (122, 142, 143). Despite the fact that PARP-1 has been reported to play a role in AP-1 activation in several inflammatory disorders, including ALI (144–146), we could not find any study in literature addressing the PARP and AP-1 interrelationship in asthma.

### PARP-1 Polymorphism in Asthma

Genetic composition of an individual plays an important role in deciding predisposition or protection against asthma (147, 148). A study in Turkish population involving 112 asthmatic patients and 180 normal controls have shown that individuals with PARP-1 762Val/Val allele were at five times higher risk of developing asthma while those with PARP-1 762Ala/Ala genotype conferred a 3.4-fold reduction in this risk, and individuals having 762Val/Ala genotype (heterozygous) conferred an even greater level of protection (149). A separate study has indicated that PARP1 Val762Ala polymorphism reduced the enzymatic activity of PARP-1 by around 40% (150). Accordingly, it is reasonable to speculate that such PARP-1 polymorphism diminishes the susceptibility to asthma by reduction in catalytic activity of the enzyme. However, we did not find other such studies on humans of different genetic background, which may prove or negate the findings of this study. Considering all, PARP-1 polymorphism may be an added risk factor in the disease development; more studies are required before we could conclude anything.

Overall, work conducted in past 14 years certainly place PARP-1 as a critical player in the orchestration of inflammatory response in asthma. We feel that future studies should consider testing PARP inhibitors in experimental models of severe asthma, a steroid non-responsive condition. Potential success of PARP inhibitors in steroid non-responsive asthma can open the venues for their clinical testing.

**Figure 2:**
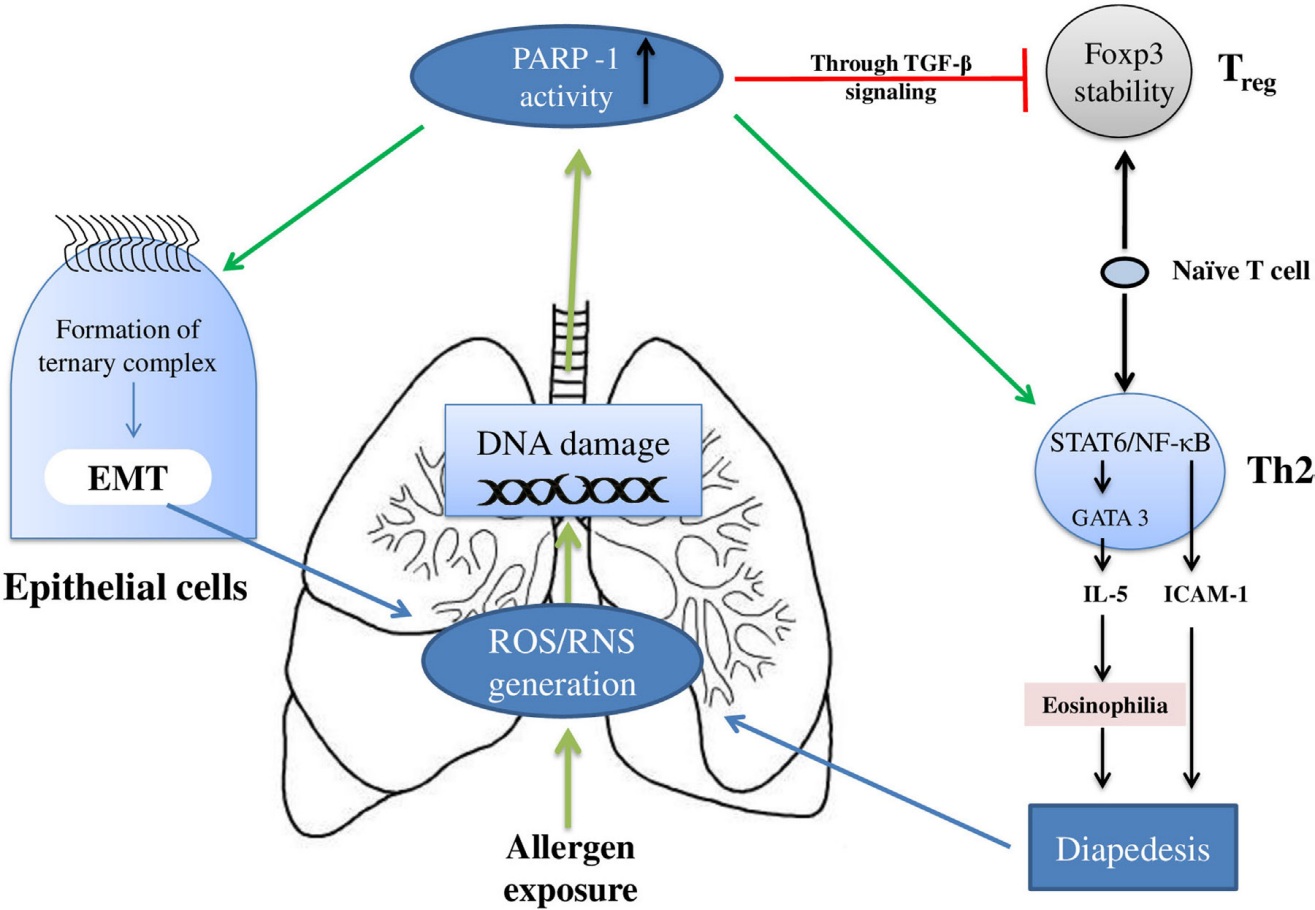
Overview of the poly(ADP-ribose)polymerase-1 (PARP-1) participation in asthma: PARP-1 primarily modulates the asthma pathogenesis by influencing the T cells and epithelial cells. In T cells, PARP-1 upregulates the clonal expansion of mature Th2 cells. In addition, PARP-1 activity reduces naïve T cells associated TGF-β signaling and FOXP3 stability; thus, eventually reducing Treg cells development. Apart from the increase in Th2 cells number, the functioning of these cells is also modulated by PARP-1 activity. PARP-1 influences transcription factors such as NF-κB and STAT-6; thus, modulating expression of cell adhesion molecules and Th2 cytokines such as IL-5. Furthermore, PARP-1 contributes in epithelial–mesenchymal transition (EMT) (part of airway remodeling) by influencing formation and binding of ternary complex.

Figure 2 summarizes the effect(s) of PARP-1 in asthma, and Table 1 presents compilation of various studies on the role of PARP-1 in asthma highlighting key finding(s) of each study.

**Table 1:** Studies on the role of poly(ADP-ribose)polymerase-1 (PARP-1) in asthma.

Study model	Mode of PARP inhibition	Effects of PARP inhibition	Significance	Reference
**ASTHMA**				

(1). *In vitro model*: H_2_O_2_ induced Human A549 cells *In vivo model*: OVA-induced mouse model *Strain*:(SV129 X C57BL/6)	*Genetic*: PARP-1^−/−^ mice *Pharmacological*: 3-AB	*In vitro model*: pharmacological inhibition ↓ LDH activity ↓ ROS generation ↓ NF-κB activation ↓ IL-8 gene expression *In vivo model*: genetic and pharmacological inhibition: ↓ Inflammatory cells recruitment (in BALF and lung tissue) ↓ PARP activation (in lung tissue) ↓ iNOS expression (in lung tissue)	First study to report the role of PARP-1 in asthma-associated airway inflammation	(32)

(2). *In vivo model*: OVA-induced guinea pig model	*Pharmacological*: 3-AB and 5-AIQ	↓ Severity of cough ↓ Occurrence of dyspnea ↓ PARP-1 activity (lung tissue) ↓ Mast cell degranulation (lung tissue) ↓ MPO activity (lung tissue) ↓ MDA level (lung tissue) ↓ TNF-α level (BALF) ↓ Nitrites level (BALF)	Reported that several allergen-induced respiratory dysfunctions are modulated by PARP-1	(70)

(3). *In vivo model*: OVA-induced mouse model *Strain*: (SV129 X C57BL/6)	*Genetic*: PARP-1^−/−^ mice *Pharmacological*: TIQ-A	Genetic and pharmacological inhibition: ↓ Eosinophil recruitment (in lung tissue and BALF) ↓ Th2 cytokine production (in lung cells): IL-4, IL-5, IL-13 ↓ OVA-specific IgE (in BALF) ↓ Mucus production (in lung tissue)	Showed that PARP-1 modulates eosinophils recruitment by influencing the expression of IL-5	(71)

(4). *In vivo model*: OVA-induced mouse model *Strain*: (SV129 X C57BL/6)	*Pharmacological*: TIQ-A	↓ Eosinophils recruitment (in BALF and lung tissue) ↓ Th2 cytokine production (in BALF cells): IL-4, IL-5, IL-13 ↓ OVA-specific IgE level (in BALF cells) ↓ Mucus production (in lung tissue) ↓ AHR	Reported the therapeutic potential of PARP inhibitors when given after allergen exposure in acute asthma	(33)

(5). *In vivo model*: OVA-induced mouse model *Strain*: (SV129 X C57BL/6)	*Genetic*: PARP-1^−/−^ mice	↓ IL-5 gene expression (in spleen) ↓ STAT-6 protein expression (in spleen) ↓ GATA-3 protein and mRNA expression (in spleen)	Explained the underlying mechanism behind PARP-1 mediated IL-5 expression. It was reported that PARP-1 activity is required for maintaining STAT6 integrity	(72)

(6). *In vivo model*: OVA-induced mouse model *Strain*: C57BL/6 and BALB/c	*Genetic*: PARP-14^−/−^ C57BL/6 mice *Pharmacological*:PJ34 (BALB/c mice used for this)	Genetic and pharmacological inhibition: ↓ Inflammatory cells recruitment (in BALF) ↓ AHR ↓ Total IgE level (in BALF and serum) ↓ OVA-specific IgE level (in serum) ↓ Th2 cytokine mRNA expression (in lung tissue): IL-4, IL-5, IL-13 ↓ Chemokine expression (in lung tissue): CCL11, CCL17, CCL24 ↓ Binding of STAT6 to Gata3 promoter	First study to show the involvement of PARP-14 in asthma pathogenesis	(132)

(7). *In vivo* model: OVA-induced guinea pig model	*Pharmacological*: HYDAMTIQ	↓ PARP-1 activation (in lung tissue and BAL cells) ↓ Severity of cough ↓ AHR ↓ Smooth muscle thickness ↓ Goblet-cell hyperplasia (in lung tissue) ↓ Collagen content (in lung tissue) ↓ MDA level (in lung tissue) ↓ MPO activity (in lung tissue) ↓ 8-hydroxy-2′deoxyguanosine level (in lung tissue) ↓ MnSOD activity (in lung tissue) ↓ Cytokines production (in lung tissue) TNF-α, IL-1β, IL-5, IL-6, IL-18 ↓ Mast cell degranulation (in lung tissue)	Reported that PARP-1 inhibition protects against some of airway remodeling traits in acute asthma	(92)

(8). *In vivo model*: OVA-induced mouse model *Strain*: (SV129 X C57BL/6)	*Genetic*: PARP-1^−/−^ mice *Pharmacological*: Olaparib	*Pharmacological and genetic inhibition*: ↓ Inflammatory cells recruitment (in BALF and lung tissue) ↓ Mucus production (in lung tissue) ↓ OVA-specific IgE (in BALF and serum) ↓ AHR ↓ Th2 cytokine production (in BAL cells): eotaxin, IL-4, IL-5, IL-13 ↓ CD4(+) T cell population (in spleen) ↑ Th1 cytokine (in BAL cells): IFN-γ ↑ Treg cell population (in spleen)	Explored the efficacy of Olaparib in blocking the already established allergic airway inflammation as well as AHR *via* modulating the CD4(+) T cell function	(73)

(9). *Clinical samples*: Human lung specimens and PBMCs *In vivo model*: HDM-induced mouse model *Strain*: (SV129 X C57BL/6)	*Genetic*: PARP-1^−/−^ mice *Pharmacological*: Olaparib	*Clinical samples*: ↑ PARP activation in PBMCs and lung tissues *in vivo model (genetic and pharmacological inhibition)*: ↓ Inflammatory cells recruitment (in BALF) ↓ AHR ↓ Th2 cytokine production (in BAL cells): eotaxin, IL-4, IL-5, IL-13 ↓ CD4(+) T cell population (in spleen) ↑ Treg cell population (in spleen)	Increased the clinical significance of previous studies as for the first time it was reported that the PARP-1 is activated in human asthma. Further, it was seen that PARP-1 inhibition effectively blocked HDM, a human allergen, induced asthma in mice	(74)

*OVA, ovalbumin; 3-AB, 3-aminobenzamide; LDH, lactate dehydrogenase; 5-AIQ, 5-aminoisoquinolinone; TIQ-A, thieno[2,3-c]isoquinolin-5-one; Treg, T regulatory; AHR, airway hyper-responsiveness; HYDAMTIQ, hydroxyl-dimethylaminomethyl-thieno[2,3-c]isoquinolin-5(4H)-one; HDM, house dust mite; MPO, myeloperoxidase activity; MDA, malondialdehyde; SOD, superoxide dismutase; PBMCs, peripheral blood mononuclear cells*.

## PARP-1 in ALI

Acute lung injury is another important pulmonary inflammatory disorder that is associated with high mortality (17, 151). Although, the pathophysiology of the disease is entirely different from asthma, neutrophilic inflammation in lungs seems to be a common factor in both ALI and steroid-resistant severe asthma. It is believed that neutrophils play a critical role in promoting steroid resistance in severe asthma (152–154). Since alternative medications are required in ALI and steroid non-responsive asthma, we next compile various studies addressing the role of PARP-1 in ALI with a focus on neutrophilic inflammation. We believe that the knowledge gathered may be applied in finding new therapies not only for ALI but also severe asthma. PARP-1 has been reported to play a key role in the manifestation of ALI induced by biological as well as mechanical insult. Accordingly, we next discuss the role of PARP-1 in ALI mediated by biological or mechanical agents separately.

### PARP-1 in Biological Insult-Induced ALI, i.e., Infection

Lipopolysaccharides-induced ALI models are the most common animal models that are used to understand the pathophysiology of the disease (155–157). These models mimic the human conditions where biological insults lead to lung injury. The various studies utilizing such models have shown that PARP-1 plays an important role in pathogenesis of ALI (138, 158, 159). Liaudet et al. have reported that PARP-1 plays a central role in LPS-mediated ALI in mice (160). Indeed, PARP-1 inhibition (genetic and pharmacological) effectively ameliorated infiltration of neutrophils in the lungs by modulating expression of several NF-κB-dependent cytokines and chemokines, NO production, and associated lipid peroxidation. Similar results were also observed in a study conducted by our group. It was observed that LPS-induced neutrophil and macrophage accumulation in lungs was reduced remarkably in PARP-1^−/−^ mice. In addition, expression of neutrophils-specific (mKC and MIP-2) and macrophage-specific (MCP-1 and MIP-1α) chemokines were found to be decreased (158). Liu et al. further explored the underlying mechanism by which PARP-1 regulates the LPS-induced airway inflammation by conducting cell culture-based study employing murine macrophages. They observed that LPS stimulation of cells enhance the binding of PARP-1 with p65 resulting in its ribosylation and ultimately increased NF-κB transcriptional activity. They have also reported that apart from DNA damage, extracellular signal-regulated kinases (ERK) pathway also play an important role in PARP-1 activation (138). The connection between PARP-1 and NF-κB signaling was further examined by Wang et al. through their *in vivo* (using LPS-induced mouse model of ALI) and *in vitro* (using mouse peritoneal macrophages) studies. They observed that PARP-1 inhibition with 3,4-Dihydro-5[4-(1-piperindinyl) butoxy]-1(2H)-isoquinoline (DPQ) blocks LPS-induced phosphorylation and degradation of IκBα, and subsequent activation of NF-κB under both *in vivo* and *in vitro* conditions (161). Veres et al. have shown that PARP inhibitor, 4-hydroxyquinazoline (4-HQN) protects against LPS-induced lung inflammation by attenuation of both NF-κB and AP-1 (162). Kiefmann et al. confirmed the earlier findings that PARP-1 mediates the LPS-induced ALI by inducing the expression of *iNOS*. However, they concluded that PARP-1 modulates the expression of *iNOS* through activation of AP-1 but not NF-κB (146).

Acute lung injury-induced secondary organ damage, specifically acute kidney injury (AKI), is another important feature of the disease that results in increased mortality (163, 164). Few studies have shown that PARP-1 inhibition is beneficial in ameliorating ALI-associated AKI. Si et al. examined the role of PARP-1 in ALI and associated AKI using rat model of LPS-induced lung injury. It was found out that PARP-1 inhibition (using 3-AB) reduces the lung inflammation *via* suppression of NF-κB activation, and restored the altered renal functions toward normal (165). Recently, we have also reported that Olaparib reduces the neutrophil infiltration and edema in lungs of LPS-administered mice potentially by downregulation of NF-κB-dependent genes such as TNF-α, IL-1β, and VCAM-1. In addition, increased serum levels of urea, creatinine, and uric acid upon LPS-mediated lung injury were restored toward normal, reflecting protective effects of the drug against ALI-associated secondary AKI (159).

### PARP-1 in Mechanical Insult Induced ALI, i.e., Ventilator-Induced Injury

Mechanical ventilation (MV) is one of the life saving therapies for patients with ALI, however, it can also damage the lungs by causing ventilator-induced lung injury (VILI) (166). VILI is an outcome of the interaction of mechanical forces with the lung structural cells, such as epithelial cells, endothelial cells, macrophages, and extracellular matrix (167). Increased permeability of endothelial and epithelial layer, air leaks, and increased level of pulmonary as well as systemic inflammatory mediators are the characteristics of VILI (168, 169). Studies have shown that repetitive mechanical stretch and shearing stress are responsible for ROS generation in endothelial cells (170–173). Also, mechanical stretch results in elevation of ROS levels in epithelial cells, which further alter cell permeability *via* activation of NF-κB (174). In 2008, Vaschetto et al. confirmed the participation of PARP-1 in VILI using rat model of the disease. They have reported that treatment with PARP-1 inhibitor, PJ-34, results in reduced lung injury, inflammatory response, and degree of apoptosis in kidneys of rats subjected to MV following LPS treatment (175). Although their experimental model is clinically relevant to the mechanism of VILI, it was difficult to identify whether this PARP-1 activation occurs because of MV, LPS, or both. Hence, uncertainty remains whether PARP-1 inhibition protect from LPS, MV, or both injuries. However, a later study resolved the issue as it was shown that PARP-1 is activated by MV itself, without the requirement of primary insult of LPS. Interestingly, they found that myeloperoxidase (MPO) activity (marker of neutrophils recruitment) was increased in response to injurious MV and correlated closely with PARP-1 activity. In addition, it was found that pre-treatment with PARP-1 inhibitor (PJ34) prevented lung injury associated with MV (176). Several studies have highlighted the importance of activation of NF-κB in VILI (177, 178). As expected, PARP-1 acts as a co-activator of NF-κB in VILI (175, 176). PARP-1 has also been linked with VILI-associated kidney dysfunction. Vaschetto et al. observed that VILI leads to kidney dysfunction *via* peroxynitrite-induced PARP-1 activation and treatment with PARP-1 inhibitor attenuates both the injuries, i.e., lung as well as kidney injury (179).

Overall, there is strong evidence that PARP-1 orchestrates the ALI-associated lung neutrophilic inflammation by modulating NF-κB activation and associated expression of several pro-inflammatory cytokines, chemokines, adhesion molecules, etc. in response to both biological and mechanical injuries. However, it is yet to be explored whether such neutrophilic inflammation can be modulated by PARP-1 inhibition in severe asthma and/or COPD.

## PARP in COPD

The persistent and progressive nature, increasing prevalence, limited pharmacological therapies, and ineffectiveness of steroids, make it is imperative to find new drug targets in COPD. Since neutrophils-associated chronic inflammation, oxidative/nitrosative stresses, and DNA damage are known to play an important role in the disease progression, there is a possibility that PARP-1 activity might be modulated during COPD pathogenesis (180, 181). Therefore, we tried to gather studies addressing the role of PARP-1 in the pathogenesis of COPD; however, we could only find a limited literature associated with PARP-1 and COPD. We used the keywords, “Cigarette smoke, COPD and PARP-1,” “PARP-1 and COPD,” “Emphysema and PARP-1,” and “Elastase induced emphysema and PARP-1” and searched Pub Med and scholarly Google articles.

Despite the fact that the role of PARP-1 in experimental COPD has not been addressed well, studies on human samples, indicate an implication of PARP-1-related activity in COPD pathogenesis. In 2003, Hageman et al. reported the systemic activation of PARP-1 in stable COPD patients. Immuno-fluorescent detection of the PAR polymers was conducted to estimate the PARP-1 activity in the peripheral blood lymphocytes. A higher percentage of PAR positive lymphocytes were observed in patients with COPD compared to the age-matched controls. Increased levels of pro-inflammatory mediators (IL-6, IL-8, and ICAM-1), reduced antioxidant capacity, uric acid, and NAD^+^ level in the plasma were also reported in patients with COPD (182). Another study conducted on PBMCs isolated from blood samples of non-smokers, non-obstructive smokers, and patients with COPD of all stages, including exacerbation, showed that levels of the DNA damage, PARP-1 activity and PARP-1 mRNA expression were found to be increased with progression of the disease. Furthermore, pre-incubation of PBMCs with UPF17 (4-methoxy-l-tyrosinyl-a-l-glutamyl-l-cysteinyl-glycine), a novel tetra peptide analog of glutathione (GSH), strongly reduced mRNA expression of PARP-1 in patients with COPD as compared to non-obstructive individuals. Interestingly, in non-obstructive smokers, higher PARP-1 activity was found as compared to patients with mild COPD (183). Overall, these two studies conducted on human samples strongly suggest occurrence of systemic oxidative stress induced DNA damage and PARP-1 activation in COPD pathogenesis.

Some *in vitro* studies have been conducted to examine the effects of PARP inhibition on cigaret smoke extract (CSE)-mediated cell death. Autophagy is a cellular process that plays a protective role by eliminating damaged proteins and organelles, thus maintains cellular homeostasis (184, 185). But prolonged and excessive autophagy might lead to cell death (186, 187). Furthermore, it has been reported that autophagy increases in COPD patients and cells exposed to CSE (185, 188, 189). To find the underlying mechanism behind CSE-induced autophagy, Hwang et al (185)., exposed human bronchial epithelial cells (H292), human fetal lung fibroblasts (HFL1), and human monocyte/macrophage cells to CSE (0.5–5%) and reported dose as well as time dependent increase in autophagy. In addition, it was shown that pre-treatment of cells H292 with Sirtuin 1 (SIRT1) activator (resveratrol) attenuated CSE-induced autophagy whereas SIRT1 inhibitor, sirtinol, augmented CSE-induced autophagy. SIRT1 is a histone deacetylase protein with physiological role in gene silencing, anti-aging, and anti-inflammation. Since, SIRT1 and PARP compete for the same substrate NAD^+^; the effects of PARP-1 inhibition on CSE-induced autophagy were also explored. It was reported that PARP-1 inhibition (with 3-AB) in HFL1 attenuates CSE-induced autophagy. The authors concluded that excessive PARP-1 activation leads to NAD^+^ depletion that causes autophagy through NAD^+^ dependent SIRT1 deactivation (185). In contradiction to abovementioned study, Kovacs et al. (190) reported survival-promoting role of PARP-1 in CSE-induced cell death. They exposed A549 lung epithelial cells to different concentration of CSE (0–20%). It was observed that CSE caused concentration-dependent cell death and impairment of proliferative capacity in A549 lung epithelial cells. Interestingly, silencing of PARP-1 or PARG using lentiviral vectors further sensitized the cells toward decreased survival as increased cell death and decreased proliferative capacity were observed at even lower concentrations of CSE. Furthermore, it was reported that delay in DNA repair was responsible for this enhanced sensitization. Overall, it was concluded that PARP-1 and PARG play a survival-promoting role in CSE-induced cell damage (190). The contradictory role of PARP-1 as observed in the two studies could possibly be due to the difference in cell lines and/or CSE concentrations that were used. In addition, it is not clear whether the survival-promoting role of PARP-1 as observed by Kovacs et al. is beneficial or detrimental. The survived cells after CSE-induced DNA damage (with PARP-1 help) may have repaired DNA completely or partially. Later case (partially repaired DNA) may render the cells prone to undesired mutations and, thus, survival-promoting role of PARP-1 may not be beneficial. Accordingly, further studies are required for better clarification.

In addition, some studies have shown the beneficial effects of several phytochemicals in COPD by exhibiting their PARP-1 inhibiting properties. Geraets et al., through their different studies reported that caffeine, its metabolites (1,7-dimethylxanthine, 3-methylxanthine, and 1-methylxanthine), and certain dietary flavonoids (flavone, myricetin, tricetin, gossypetin, delphinidin, quercetin, and fisetin) have PARP-1-inhibiting properties (191–193). Furthermore, they evaluated the protective effects of flavone, fisetin, morin, or tricetin on expression of pro-inflammatory cytokines, such as IL-6 and TNF-α, using an *ex vivo* system after procurement of blood samples from healthy men and patients with COPD and type 2 diabetes (T2D). In whole blood assays, samples from each participant were exposed to 1 µg/L LPS with or without pre-incubation with 10 µmol/L of flavone, fisetin, morin, or tricetin. It was observed that pre-incubation with fisetin and tricetin, reduced the LPS-induced TNF-α and IL-6 production in all groups with more pronounced effects in patients with COPD and T2D (194). In another study, anti-inflammatory effects of a previously stated PARP-1 inhibitor, i.e., 1, 7-dimethylxanthine was also reported using both mouse model as well as *ex vivo* system utilizing blood samples from COPD patients. It was found that 1,7-dimethylxanthine significantly attenuated MPO activity, cytokine (IL-6, TNF-α, MIP-1α, and MIP-2) production, and PAR polymer formation in LPS-exposed mouse model. Under *ex vivo* conditions too, 1,7-dimethylxanthine mitigated the production of cytokines (IL-6 and TNF-α) in LPS-exposed blood samples of patients with COPD (195). Considering the PARP-1 inhibiting effects of the abovementioned compounds (flavonoids and caffeine metabolites), there is a possibility that PARP-1 activity may directly or indirectly influence the pro-inflammatory cytokine production in COPD. Given the fact that PARP-1 has ability to alter NF-κB activation and that several pro-inflammatory factors involved in COPD pathogenesis are dependent on activation of NF-κB, we speculate that decreased levels of TNF-α and IL-6 upon PARP inhibition as observed by Geraets et al. might be through suppression of NF-κB activation.

Nitrosative stress is another factor that has been associated strongly with COPD pathogenesis. Numbers of studies have shown that nitrosative stress plays an important role in the disease and might be linked with steroid resistance (153, 196, 197). Interestingly, Seimetz et al. (198) have reported that *iNOS* inhibition prevented tobacco-smoke-induced structural and functional alterations of both the lung vasculature and alveoli and even reversed the already established disease (198). Since the expression of *iNOS* is reported to be modulated by PARP-1, the protein may contribute to COPD pathogenesis *via iNOS* dependent nitrosative stress (49). Accordingly, it would be reasonable to explore the contribution of PARP-1 in nitrosative stress mediated COPD pathogenesis.

Altogether, the abovementioned studies strongly suggest that the PARP-1 activity is modulated during the pathogenesis and progression of COPD. However, evidence is meager to draw complete conclusions about the role of PARP-1 in the disease pathogenesis. Most of the studies have been conducted on patient blood samples, and none of them have used established animal models of COPD. Moreover, the available potent PARP inhibitors have not been used in any of the studies to evaluate effects of PARP inhibition on the disease progression. Hence, further studies are required to clarify the role of PARP in COPD using both *in vitro* and *in vivo* experimental systems. Table 2 summarizes the findings of various studies on the role of PARP in ALI and COPD.

**Table 2:** Studies on the role of poly(ADP-ribose)polymerase-1 (PARP-1) in acute lung injury (ALI) and chronic obstructive pulmonary disease (COPD).

Study model	Mode of PARP inhibition	Effects of PARP inhibition	Significance	Reference
**ALI**				

(1). *In vivo model*: LPS-induced mouse model *Strain*: Balb/c and C57BL/6	Genetic: PARP-1^−/−^ C57BL/6 mice *Pharmacological*: PJ-34 (BALB/c mice used for this)	*Pharmacological and genetic inhibition*: ↓ Neutrophil recruitment (in BALF) ↓ Cytokines level (in BALF): TNF-α, IL-6, IL-1β ↓ Chemokines level (in BALF): MIP-1α, MIP-2 ↓ NO production (in BALF) ↓ MPO activity (in BALF) ↓ MDA level (in lungs) ↓ Lung injury	Showed the crucial role of PARP-1 in LPS-induced pulmonary inflammation	(160)

(2). *In vivo model*: LPS-induced rabbit model	*Pharmacological*: 3-AB	↓ PARP activation (in lung tissue) ↓ iNOS protein and mRNA expression (in lung tissue) ↓ Plasma nitrite concentration ↓ MDA level (in plasma and lung tissue) ↓ AP-1 activation (in lung tissue)	Reported that PARP-1 modulates iNOS expression (*via* activation of AP-1 not NF-κB) during LPS-induced ALI	(146)

(3). *In vivo model*: Ventilator-induced mouse model *Strain*: C57BL/6	*Pharmacological*: PJ 34	↓ Lung injury score ↓ PARP activation (in lung tissue) ↓ MPO activity (in BALF) ↓ NO concentration (in BALF) ↓ Cytokines level (in BALF): TNF-α, IL-6 ↓ NF-κB-DNA binding (in lung tissue)	Confirmed the involvement of PARP-1 in pathogenesis of VILI (induced without any LPS priming)	(176)

(4). *In vivo model*: LPS-induced rat model *Strain*: Sprague-Dawley rat	*Pharmacological*: 3-AB	↓ Plasma levels of lactate, creatinine and potassium ↓ Cytokine mRNA expressions: TNF-α, IL-1β, IL-6 (lung and kidney tissue) ↓ PARP protein expression (lung and kidney tissue) ↓ NF-κB protein expression (lung and kidney tissue) Preserved the renal function	PARP-1 inhibition protected against ALI-associated AKI by affecting expression of pro-inflammatory cytokines dependent on NF-κB activation	(165)

(5). *In vivo model*: LPS-induced mouse model *Strain*: C57BL/6 *In vitro model*: Peritoneal macrophages isolated from mice	*Pharmacological*: DPQ	↓ Neutrophils recruitment (in lung tissue) ↓ MPO activity (in lung tissue) ↓ Cytokines mRNA expression (in lung tissue) TNF-α, IL-1β, IL-6 ↓ Chemokines mRNA expression (in lung tissue) MIP-2, CXCL-1 ↓ iNOS gene expression (in lung tissue) ↓ Vascular permeability ↓ Degree of apoptosis (in lung tissue) ↓ Degradation of IkB-α (in lung tissue) ↓ NF-kB activation (in lung tissue)	Showed that PARP-1 plays a critical role in LPS-induced inflammation *via* modulation of NF-κB signaling	(161)

**COPD**				

(1). *Clinical samples*: plasma and PBMCs isolated from COPD patient’s blood	–	↑ Percentage of PAR polymer-positive lymphocytes ↑ Cytokine level (in Plasma): IL-6, IL-8 ↑ Adhesion molecule level (in Plasma): ICAM-1 ↓ Plasma TEAC ↓ Plasma uric acid ↓ Blood and lymphocyte NAD level	First report to highlight that PARP-1 activity is systematically increased during the pathogenesis of COPD in humans	(182)

(2). *In vitro model*: (1). MNNG-treated *A549* human lung epithelial cells (2). LPS-treated *RF24* Vascular endothelial cells	By using Flavones	*In MNNG-treated A549 and RF24*: ↓ PARP-1 activity ↓ NAD level ↓ PAR polymers formation *In LPS-treated A549*: ↓ IL-8 production ↑ IκBα transcription	Explored the PARP inhibiting properties of flavones	(193)

(3). *In vivo model*: LPS-induced mouse model *Strain*: C57BL/6 *Ex vivo* model: Blood samples of healthy control and COPD patients	*Pharmacological*: caffeine metabolite: 1,7-dimethylxanthine	*In vivo model*: ↓ MPO activity (in lung tissue) ↓ Cytokines mRNA expression (in lung tissue): TNF-α, IL-6 ↓ Chemokines mRNA expression (in lung tissue): MIP-1α, MIP-2 ↓ Plasma IL-6 level ↓ IκBα transcription (in lung tissue) ↓ PAR polymer formation (in lung tissue) *ex vivo model*: ↓ LPS-induced production of cytokine: TNF-α, IL-6	Explored the therapeutic potential of PARP-1 inhibiting caffeine metabolite	(161)

(4). *Clinical samples*: PBMCs isolated form COPD patient’s blood	*By using UPF17*, Tetrapeptide antioxidant	↓ PARP-1 mRNA expression	Showed that the DNA damage and subsequent PARP-1 activity is increased during the progression of COPD	(183)

*LPS, lipopolysaccharide; 3-AB, 3-aminobenzamide; DPQ, 3, 4-Dihydro-5[4-(1-piperindinyl)butoxy]-1(2H)-isoquinoline; TEAC, Trolox equivalent antioxidant capacity; MNNG, N-methyl-N′-nitro-N-nitrosoguanidine; PAI-1, plasminogen activator inhibitor; VILI, ventilator-induced lung injury; AKI, acute kidney injury; MPO, myeloperoxidase activity; MDA, malondialdehyde; PBMCs, peripheral blood mononuclear cells*.

## Conclusion

**Figure 3:**
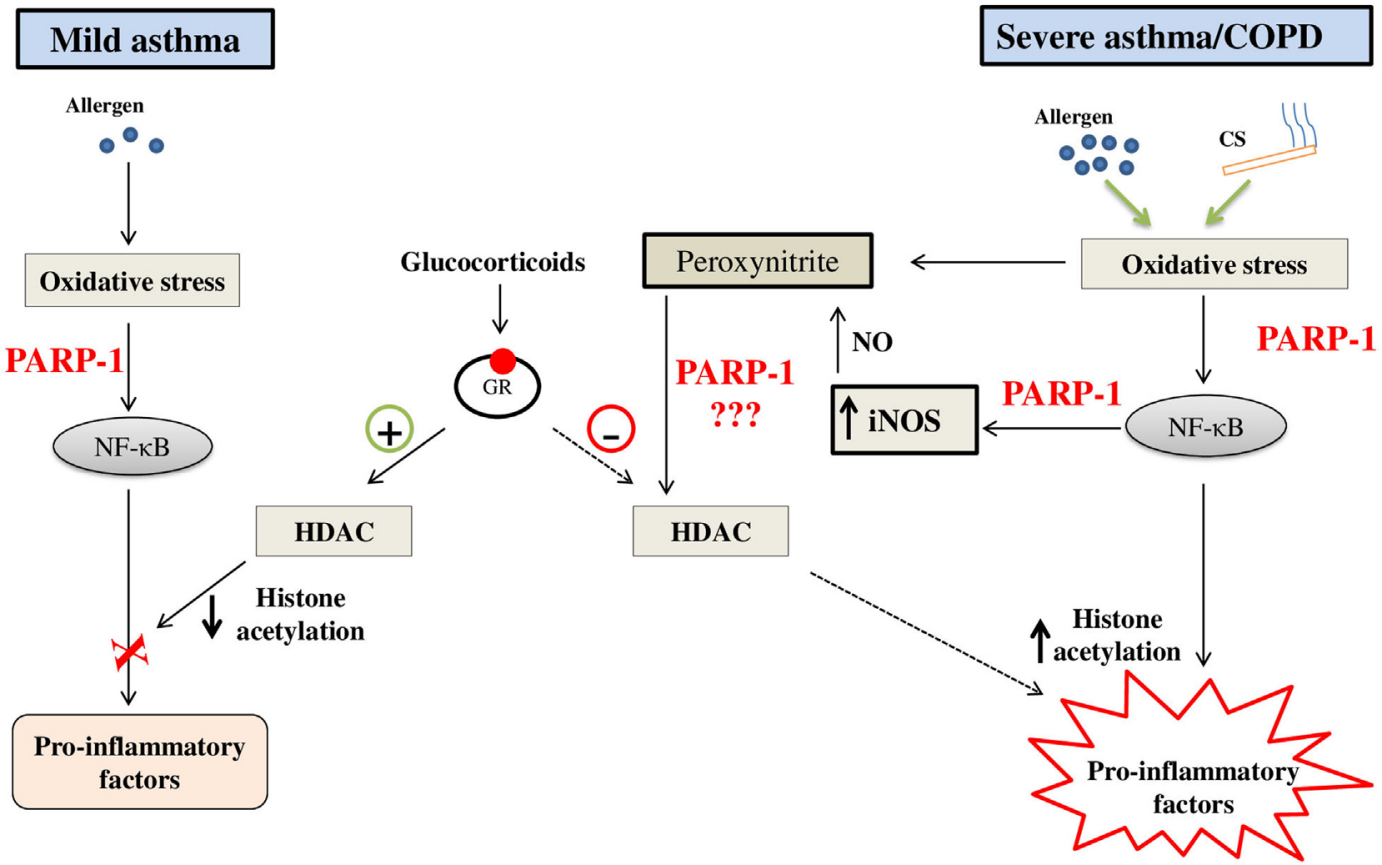
A potential model on the role of poly(ADP-ribose)polymerase-1 (PARP-1) in steroid responsive mild asthma *vis-à-vis* steroid-resistant severe asthma/chronic obstructive pulmonary disease (COPD): PARP-1 may contribute to the steroid resistance by generation of excessive nitrosative stress through enhanced and persistent expression of inducible nitric-oxide synthase (*iNOS*) due to infiltration of neutrophils. Nitrosative stress ultimately results in nitration of proteins such as histone deacetylases (HDACs) and causes steroid resistance through their altered functions (hampered deacetylation of histones). PARP-1 inhibition may restore the function of HDAC2 by reducing their nitration and, thus, ameliorates expression of pro-inflammatory genes in severe asthma and COPD. Abbreviations: CS, cigaret smoke; NO, nitric oxide.

Based on the outcome of preclinical studies, we can safely say that PARP-1 inhibition is a beneficial strategy to block lung inflammation at least in asthma and ALI. However, clinical efficacy of the strategy focusing on PARP-1 inhibition in humans afflicted with lung inflammatory disorders remains to be tested. The steroids are the most efficacious therapy for asthma till date. Considering the side effects of steroids and their lack of effectiveness in severe asthma and COPD, the therapeutic potential of new drug candidates in the area must be tested. It is important to note that primary inflammation associated with the moderate asthma is eosinophilic; while, in severe asthma and COPD, it is neutrophilic in nature. Incidentally, neutrophils have been critically linked with steroid resistance in asthma and COPD. Therefore, targeting steroid resistance neutrophilic inflammation in chronic lung pathologies would be a logical extension of previously reported work. Accordingly, it would be interesting to test the PARP-1 inhibitors (alone or in combination with steroids) in chronic respiratory disorders such as severe asthma and COPD with an aim to block recruitment of neutrophils into the airways. In view of the studies reported by us and others that PARP-1 inhibition protects against not only eosinophilic inflammation but also against lung neutrophilia by blocking activation of NF-κB (pro-inflammatory transcription factor), the chances of success of these compounds in treatment/prevention of severe form of asthma and/or COPD would be high (Figure 3 depicts potential model). Once these compounds pass this preclinical phase, it may open up new avenues for translational research in the area.

## Author Contributions

GSS and VD have contributed equally to this paper. GSS and VD have compiled various studies and wrote the paper. ASN is the principal investigator who has conceptualized the idea of review paper, contributed in preparation of final draft and training of co-authors.

## Conflict of Interest Statement

The authors declare that the research was conducted in the absence of any commercial or financial relationships that could be construed as a potential conflict of interest.
